# Spatiotemporal disparities and dynamic transition characteristics of China’s national standard development contribution levels—Based on machine splitting and location assignment technology

**DOI:** 10.1371/journal.pone.0327002

**Published:** 2025-09-09

**Authors:** Hongjun Sun, Yang Du, Juxiu Huang

**Affiliations:** 1 China National Institute of Standardization, Beijing, China; 2 Biaoxin Science & Technology (Beijing) Co., Ltd, Beijing, China; Lanzhou Jiaotong University, CHINA

## Abstract

This study examines China’s national standard development from 2001 to 2023. Using machine splitting and location assignment technology, the Dagum Gini coefficient and its decomposition methods, and traditional and spatial Markov chain estimation methods, we identify the spatiotemporal disparities and dynamic transition characteristics of the contribution levels to national standard development across China’s eight comprehensive economic zones. The findings provide a reference for promoting regional coordinated sustainable development and high-quality economic transformation. The study reveals three key findings. (1) Contribution levels of China’s eight comprehensive economic zones to national standard development have significantly increased. The Northern Coastal comprehensive economic zone has the highest contribution levels, followed by the Eastern and Southern Coastal zones, whereas the Northwestern and Northeastern zones have lower contribution levels. (2) The overall regional disparity in national standard development contribution levels is decreasing, with the largest intraregional disparities found in the Northern and Southern Coastal zones. Significant interregional disparities persist between the Northern Coastal and Northwestern zones, the Eastern Coastal and Northwestern zones, and the Southern Coastal and Northwestern zones, with interregional disparities being the primary driver of the overall regional gap. (3) When spatial correlation effects are not considered, contribution levels exhibit clear signs of club convergence and asymmetric distribution. However, when spatial correlation effects are considered, the transition characteristics of contribution levels show significant spatial dependence. This study makes three key contributions. First, it illustrates the spatiotemporal differentiation and dynamic transition characteristics of the contribution levels of China’s eight comprehensive economic zones to national standard development, addressing gaps in existing quantitative research. Second, it introduces novel techniques and decomposition methods. Third, it identifies the primary causes of regional disparities and dynamic transition characteristics, providing empirical evidence to support policy decisions for regional coordinated sustainable development and high-quality economic transformation.

## Introduction

National standards are strategic innovation resources that comprehensively reflect scientific technology and practical experience, representing the level of technological innovation and industrial development across various sectors. As China’s economy enters a new era of innovation-driven, high-quality development, advancing this development through high standards has become a central focus. New economic geography and standard economics theories suggest that in this new era, regional economic high-quality development must emphasize industrial growth while increasingly prioritizing knowledge creation, technological innovation, and standard development [1–10]. National standards, approved and published by China’s national standardization authority, play a crucial role in economic and technological development, particularly in guiding and coordinating industrial advancement and technological innovation activities [11,12]. These standards reflect a commitment to achieving national development goals and are applied uniformly across the country.

Regional development is a key factor in achieving high-quality economic growth, with most regions in China integrating standard development as a vital component of their innovation-driven development strategies. National standard development has increasingly become a major driving force in transforming regional development models and fostering high-quality regional development. However, in this phase of high-quality development, social contradictions have shifted toward the conflict between rising demands for a better quality of life and unbalanced, insufficient development. Against this backdrop, disparities in regional contributions to national standard development have become a pressing issue, posing challenges to the coordinated advancement of industry and innovation across regions.

This study seeks to answer a fundamental question: do the contribution levels of China’s eight comprehensive economic zones to national standard development exhibit significant spatiotemporal differentiation and dynamic transition characteristics, and how do regional disparities evolve over time? According to the *Annual Report on Standardization Development of China* (2023), as of the end of 2023, China had 44,499 national standards and 4,164 national standard samples [13]. These standards are developed by diverse drafting entities and have widespread geographical distribution. A major challenge addressed in this study is how to scientifically and systematically evaluate a region’s contribution level to national standard development. Unlike other countries, China’s national standards list the drafting units, which are spread and applied across various regions. This unique characteristic enables the construction of a regional national standard development contribution index based on the national standards drafted by entities in each region, similar to the method used to assign patents or scientific papers to specific regions. This approach provides the foundation for employing machine splitting and location assignment technology to evaluate regional standard development contributions. Thus, this study measures the contribution levels of China’s eight comprehensive economic zones and examines their spatiotemporal differentiation and dynamic transition characteristics, aiming to reveal spatial disparities in and evolutionary patterns of these contribution levels. The key innovations and contributions of this study are described below.

First, this paper provides methodological and technical support for future research on regional contributions to national standard development. National standards summarize scientific and practical experiences, thereby signaling the quality of regional economic development. Different methodologies and models yield varying results when calculating the regional national standard development contribution index, making the establishment of a scientifically valid and reasonable evaluation method a common challenge in this field. Therefore, this study focuses on national standards as the primary research object. In the early stages of this study, various models were tested and repeatedly simulated based on the actual development of China’s standards. Ultimately, machine splitting and location assignment technology were introduced to construct the regional national standard development contribution index in order to obtain more reliable results that reflect contribution levels of different regions. This methodological advancement provides a valuable reference for future studies on standard development contribution indices and related research processes.

Second, this paper contributes to the practical application of the analysis of regional contribution levels to national standard development as a policy tool. Currently, research on the regional national standard development contribution index is limited, particularly regarding regional disparities and the dynamic evolution of contribution levels across China’s eight comprehensive economic zones. Existing studies mainly focus on national or industry-level analysis, often providing qualitative descriptions of the overall performance in terms of the number of standards issued. However, quantitative analyses of regional disparities and dynamic transition characteristics in national standard development contribution levels across the eight comprehensive economic zones are generally lacking. There remains a gap between current research and its application in guiding the actual development of standards across different regions. To address this gap, this paper employs the Dagum Gini coefficient and its decomposition method to measure and decompose regional disparities in national standard development contribution across China’s eight comprehensive economic zones. Additionally, spatial Markov chain estimation methods are used to examine the dynamic transition characteristics of contribution levels. This approach enables a more systematic and in-depth study of spatial imbalances and evolution trends, filling gaps in both theoretical and applied research and effectively informing decision-making processes.

Third, the findings of this study help generate a more accurate understanding of the current spatial imbalance in national standard development contributions and identify key factors for promoting regional collaborative sustainable development. The conclusions provide a detailed assessment of spatial imbalances and dynamic evolutionary trends in China’s standardization landscape. Based on these insights, practical policy recommendations can be formulated to address current issues of spatial imbalance and regional sustainability. This research serves as a valuable tool for policymakers to both understand and solve related problems.

This paper is structured as follows: Section 1 presents the literature review and problem statement, Section 2 discusses the research methods and data sources, Section 3 presents the measurement results of the national standard development contribution index in the eight comprehensive economic zones, Section 4 presents the results of the Dagum Gini coefficient and its decomposition, Section 5 discusses the dynamic transition characteristics of national standard development contribution levels across the eight comprehensive economic zones, and Section 6 presents the conclusions and discussion.

## Section 1: Literature review and problem statement

Standard economics theory [14,15] suggests that standards drive economic growth by fostering economies of scale, effective division of labor, development of specialized capabilities, network effects, reduced barriers and costs, and enhanced trade trust. It argues that standards, alongside capital, labor, and technology, constitute a crucial factor underpinning economic growth. New economic geography theory [16,17] posits that disparities in capital, labor, technology, and standards create spatial spillover effects, leading to imbalances that drive regional disparities in economic growth [18–20]. Thus, analyzing the spatiotemporal differentiation and dynamic transition characteristics of national standard development contributions can help clarify the role of standards in economic growth and also provide insights into regional disparities and imbalance in China’s economic development. A review of domestic and international literature reveals that current research primarily focuses on four key areas.

First, research on the selection of research objects and the measurement of regional contributions to national standard development has largely focused on industry-specific comparisons of national standards, such as those in smart construction [21–24] and food safety [25–28], across countries. However, contribution levels to national standard development at the regional level within a single country have remained underexplored. Moreover, no standardized method has been established to measure the contribution index of national standard development across regions in China. This is largely due to the wide distribution of national standards, which makes it difficult to accurately assign them to specific regions [29–31]. To address this gap, this study examines the national standard development contribution index across China’s eight comprehensive economic zones. In the preliminary stages, various models were selected and repeatedly tested based on the actual development of China’s national standards. To the best of our knowledge, this research is the first to propose the use of machine splitting and location assignment technology to construct the national standard development contribution index at the regional level. This approach introduces two key innovations. First, unlike other countries’ national standards, China’s national standards explicitly list the drafting units, which are spread across regions. This method constructs a regional contribution index based on all national standards drafted by units in each region, fully reflecting the unique characteristics of China’s national standards development process. Furthermore, this method assigns scores to each region by analyzing the ranking or position of drafting units in national standards from different regions. This enables a more comprehensive assessment of the quantity and quality of regional contributions to national standard development, providing a precise characterization of contribution levels across regions.

Second, research on regional disparities in national standard development contributions has primarily relied on direct data comparisons between countries [32,33]. However, this approach is highly susceptible to subjective bias and loses accuracy as the sample size increases, leading to significant errors and ineffective decision-making support [34]. To improve measurement accuracy, methods such as the coefficient of variation and Theil index [35–38] have been applied. The coefficient of variation can not accurately decompose intraregional and interregional disparities and effectively reflect the sources of regional disparities. Although Theil index can decompose the regional gap into intraregional and interregional gap, it does not consider the distribution of subsamples and the contribution of cross-terms, which leads to the deviation of the calculation results. To address these shortcomings, this study introduces the Dagum Gini coefficient and its decomposition methods [39–45] to determine overall regional disparities in national standard development contributions and to identify their primary sources. This method can decompose regional disparities into intraregional and interregional components while considering the distribution of subsamples and the contributions of interaction terms; thus, it serves as a more comprehensive and precise analytical tool compared with intuitive comparisons, the coefficient of variation, or the Theil index.

Third, research on the dynamic transition characteristics of regional national standard development contributions has primarily used intuitive comparison methods to examine changes over time [46–49]. These studies visually compare trends in national standard development contributions across different regions to qualitatively assess their dynamic evolution. To reduce the subjective bias associated with intuitive comparisons, the Kernel density estimation method has been used to quantitatively reveal the dynamic transition characteristics of research samples [50–52]. However, Kernel estimation is ineffective in capturing the spatial evolution and transition characteristics of research samples within the context of spatial association effects. In the case of this study, spatial association effects inevitably influence the spatial transition characteristics of national standard development contributions. For instance, as the coordinated development strategy of Beijing, Tianjin, and Hebei evolves, the frequency and intensity of exchanges and cooperation among these three regions has increased. This has strengthened Beijing’s knowledge spillover effects on Tianjin and Hebei in national standard development, naturally increasing these two regions’ contributions and reducing regional disparities. The findings presented later in this paper support this view. In recent years, the spatial Markov chain estimation method has been used to analyze the dynamic evolution of variables under spatial association effects [53,54]. To objectively examine the impact of spatial association effects on dynamic transition characteristics, this study constructs a spatial weight matrix and employs traditional and spatial Markov chain estimation methods [55–57] to explore the dynamic transition characteristics of national standard development contributions across China’s eight comprehensive economic zones from 2001 to 2023.

Fourth, research on policy recommendations for coordinated regional development of national standard development contribution has gained increasing attention. However, current studies remain limited in both breadth and depth [58–60]. Most research focuses on qualitative analyses of standardization challenges and presents policy suggestions that are often subjective, lacking scientific, systematic, and practical application. To address these shortcomings, this study adopts a multidimensional quantitative approach to explore the spatiotemporal differentiation and dynamic transition of national standard development contributions. This approach enables the generation of practical policy recommendations for reducing regional imbalances and promoting coordinated regional development, thereby enhancing the practical application value of this research. The results provide empirical evidence for regional coordination efforts and further enrich the theoretical framework on collaborative improvements in national standard development capacity.

The key questions this study addresses are as follows: Do the contribution levels of national standard development in China’s eight comprehensive economic zones exhibit significant spatiotemporal differentiation and dynamic transition characteristics? What are the overall, intraregional, and interregional disparities, and how do they contribute to total disparity? Are these disparities widening or narrowing over time, and what are their primary sources? What spatial transition characteristics and evolution patterns can be observed? What policies should be implemented to promote the coordinated improvement of national standard development contributions across regions? By answering these questions, this study provides a quantitative assessment of current contribution levels to national standard development in China’s eight comprehensive economic zones while advancing the understanding of regional imbalances. Moreover, the analysis of spatiotemporal transition characteristics and distribution patterns offers important theoretical and practical insights for fostering the coordinated and collaborative development of national standards across regions.

## Section 2: Research methods and data sources

### Research methods

#### Machine splitting and location assignment technology.

The regional standard development contribution index measures a region’s contribution to national standard development. This study quantifies the contribution levels of China’s eight comprehensive economic zones using machine splitting and location assignment technology. Contribution values are assigned based on the order in which drafting units appear in national standard documents. By leveraging the Baidu Maps API, the values of all drafting units within a given region are aggregated to generate that region’s national standard development contribution index. The machine splitting and location assignment process comprises the following steps:

A combination of manual and automated extraction methods is used to compile information on all drafting units involved in developing national standards. A database of drafting units is constructed using metadata such as standard numbers, standard names, drafting unit names, and ranking order.

Then, experts establish assignment rules (as shown in Table 1), and the machine automatically assigns values *u* based on the order in which drafting units appear in national standard documents.

**Table 1:** Contribution Levels Assignment Table for National Standard Development.

Rank	1st	2^nd^	3rd	4th	5th	6th or lower
*u*	1	0.8	0.6	0.4	0.2	0.1

The Baidu Maps API is used to determine the geographic locations of different drafting units. The contributions of all drafting units within the same region are then summed to calculate the regional national standard development contribution index.

The contribution index I  for a drafting unit in a specific region is defined as follows:


$$\begin{array}{c} I_{s}=\sum {\mathrm{\backslash nolimits}}_{q=1}^{n}u_{q} \end{array}$$ (1)

where $I_{s}$ represents the national standard development contribution index of a drafting unit within region *s*. Variable n is the total number of national standards drafted by a unit within region *s*, and *u*_*q*_ is the contribution score of a drafting unit in region *s* for the *q*-th national standard.

The regional national standard development contribution index A aggregates the contribution scores of all drafting units within a given region. It is calculated using the following formula:


$$\begin{array}{c} A_{s}=\sum {\mathrm{\backslash nolimits}}_{e=1}^{m}I_{s} \end{array}$$ (2)

where $A_{s}$ represents the regional standard development contribution index for region *s*, *I*_*s*_ the national standard development contribution index for a drafting unit within region *s*, and *m* is the total number of drafting units in region *s*.

#### Dagum Gini coefficient and its decomposition method.

The Dagum Gini coefficient and its decomposition method are used to measure and analyze regional disparities in the national standard development contribution levels across China’s eight comprehensive economic zones. The Dagum Gini coefficient is defined by the following equation:


$$G=\frac{\sum_{j=1}^{k}\sum_{h=1}^{k}\sum_{i=1}^{n_{j}}\sum_{r=1}^{n_{h}}\left|y_{ji}-y_{hr}\right|}{2n^{2}\overset{―}{Y}}$$ (3)

$$\overset{―}{Y_{h}}\leq \cdots {\overset{―}{Y}}_{j}\leq \cdots {\overset{―}{Y}}_{k}$$ (4)

In Eq. (3), G represents the overall Gini coefficient, where a higher G indicates greater regional disparities in the national standard development contribution index. The number of regions, *k*, refers to the eight comprehensive economic zones analyzed in this study: Northeast, Northern Coastal, Eastern Coastal, Southern Coastal, Yellow River Middle Reaches, Yangtze River Middle Reaches, Southwest, and Northwest. Variables i and r represent the number of provinces or cities within a region. The term $n_{j}\left(n_{h}\right)$ denotes the number of provinces or cities in region j(h), and $y_{ji}\left(y_{hr}\right)$ refers to the contribution level of any province or city in region j(h). The total number of provinces or cities is represented by n, with the average contribution level calculated as the mean of all provinces or cities. Note that when using the Dagum Gini coefficient decomposition method, regions must first be ranked based on their average contribution levels, as shown in Eq. (4) above.


$$G_{jj}=\frac{\frac{1}{2{\overset{―}{Y}}_{j}}\sum_{i=1}^{n_{j}}\sum_{r=1}^{n_{j}}\left|y_{ji}-y_{jr}\right|}{{n^{2}}_{j}}$$ (5)

$$G_{w}=\sum_{j=1}^{k}G_{jj}p_{j}s_{j}$$ (6)

$$G_{jh}=\frac{\sum_{i=1}^{n_{j}}\sum_{r=1}^{n_{h}}\left|y_{ji}-y_{hr}\right|}{n_{j}n_{h}({\overset{―}{Y}}_{j}+\overset{―}{Y_{h}})}$$ (7)

$$G_{nb}=\sum_{j=2}^{k}\sum_{h=1}^{j-1}G_{jh}(p_{j}s_{h}+p_{h}s_{j})D_{jh}$$ (8)

$$G_{t}=\sum_{j=2}^{k}\sum_{h=1}^{j-1}G_{jh}(p_{j}s_{h}+p_{h}s_{j})(1-D_{jh})$$ (9)

$$D_{jh}=\frac{d_{jh}-p_{jh}}{d_{jh}+p_{jh}}$$ (10)

$$d_{jh}=\int_{0}^{\infty }dF_{j}(y)\int_{0}^{y}\left(y-x\right)dF_{h}(x)$$ (11)

$$p_{jh}=\int_{0}^{\infty }dF_{h}(y)\int_{0}^{y}\left(y-x\right)dF_{j}(x)$$ (12)

Equations (5) and (6) define the Gini coefficient $G_{jj}$ within region *j* and its intraregional contribution rate *G*_*w*_. Equations (7) and (8) define the interregional Gini coefficient *G*_*jh*_ between regions *j* and *h*, in addition to the interregional contribution rate *G*_*nb*_. Equation (9) defines the contribution rate of super-variable density *G*_*t*_, where *p*_*j*_* = n*_*j*_*/n*, *(j = 1,2,∙∙∙,k)*. Equation (10) defines the relative influence of interregional contribution *D*_*jh*_ between regions j and h. $d_{jh}$ is calculated as the mathematical expectation of the sum of all sample values where *y*_*hr*_* − y*_*ji*_ > 0 for regions *j* and h. In Eqs. (11) and (12), F_*h*_ and *F*_*j*_ represent the cumulative density distribution functions of regions *h* and *j*, respectively.

#### Traditional and spatial Markov chain estimation methods.

The Markov chain estimation method is used to analyze the spatial evolution characteristics of national standard development levels across various regions during different sample periods by constructing a Markov transition probability matrix. This method represents a stochastic process, denoted as {*X*_*a*_*, a ∈ A*}, where the values belong to a finite set *M*, with the elements of this set representing the states of the random process. Index set *A* corresponds to different time periods. Let the random variable *X*_*b*_* = j*, which indicates that the system is in state *j* during period *b*, and the Markov property is satisfied as expressed in Eq. (13). The key characteristic of the Markov chain is that the conditional distribution of *X*_*b*_ depends only on the state *X*_*b-1*_. Suppose *P*_*ij*_ represents the probability of a region transitioning from state *i* in year *b-1* to state *j* in year b. As a result, $P_{ij}=n_{ij}\text{/}n_{i}$, where $n_{ij}$ is the number of regions that transitioned from state *i* in year *b-1* to state *j* in year *b*, and *n*_*i*_ is the number of regions in state *i* in year *b-1*.


$$P=\left\{X_{b}=j\;\right|\;X_{b-1}=i_{b-1},X_{b-2}=i_{b-2},\ldots ,X_{0}=i_{0}\}=P\left\{X_{b}=j\;\right|\;X_{n-1}=i\}=P_{ij}$$ (13)

If the national standard development contribution levels in the eight comprehensive economic zones are evenly divided into *N* grades, an *N×N* transition probability matrix can be constructed. This allows for an analysis of the spatial transfer characteristics and patterns of national standard development contribution levels across the eight comprehensive economic zones from a transition probability perspective.

Spatial Markov chain analysis extends this approach by incorporating spatial association into the analysis, transforming the N × N transition probability matrix into an *N× N × N* transition probability matrix. In this context, $P_{ij}$ represents the probability of transitioning from grade *i* in year *t* − 1to grade *j* in year *t*, given that the region’s spatial lag grade in year *t* − 1 is *N*_*i*_. This approach enables a sys*t*ematic evalua*t*ion of the impact of spatial correlation effec*t*s on the spatial evolution of national standard development contribution levels across the eight comprehensive economic zones.

Because the samples represent the national standard development contribution levels of the eight comprehensive economic zones, this study constructs a spatial weight matrix *W*_*mn*_ based on whether provinces or cities share a border. If province *m* shares a border with province *n*, then ${\tilde{w}}_{mn}=\;1;$ otherwise ${\tilde{w}}_{mn}=\;0$. On this basis, row standardization is performed by calculating the ratio of each matrix element ${\tilde{w}}_{mn}$ to the sum of the elements in its row, as expressed in Eqs. (14) and (15). The spatial lag value is then calculated using the spatial weight matrix, which spatially weights the national standard development contribution index of neighboring regions, as shown in Eq. (16).


$$w_{mn}\equiv \frac{{\tilde{w}}_{mn}}{\sum_{n}{\tilde{w}}_{mn}}$$ (14)

$$W_{mn}=(\begin{array}{ccc} w_{11} & \cdots & w_{1n}\\ \vdots & \ddots & \vdots \\ w_{m1} & \cdots & w_{mn} \end{array})$$ (15)

$$Lag={\mathrm{\backslash sumW}}_{mn}X_{t}$$ (16)

### Data sources

The data for this study are primarily sourced from the National Standards Library of the China National Institute of Standardization, covering all national standards data from 2001 to 2023. The division of China’s eight comprehensive economic zones is presented in Table 2.

**Table 2:** Region Division.

Comprehensive Economic Zone	Covered Regions	Comprehensive Economic Zone	Covered Regions
Northeast	Liaoning, Jilin, Heilongjiang	Yellow River Middle Reaches	Shaanxi, Shanxi, Henan, Inner Mongolia
Northern Coastal	Beijing, Tianjin, Hebei, Shandong	Yangtze River Middle Reaches	Hubei, Hunan, Anhui, Jiangxi
Eastern Coastal	Shanghai, Jiangsu, Zhejiang	Southwest	Yunnan, Guizhou, Sichuan, Chongqing, Guangxi
Southern Coastal	Fujian, Guangdong, Hainan	Northwest	Gansu, Qinghai, Ningxia, Tibet, Xinjiang

## Section 3: Measurement results of the national standard development contribution index in the eight comprehensive economic zones

### Overall trend analysis

**Fig 1:**
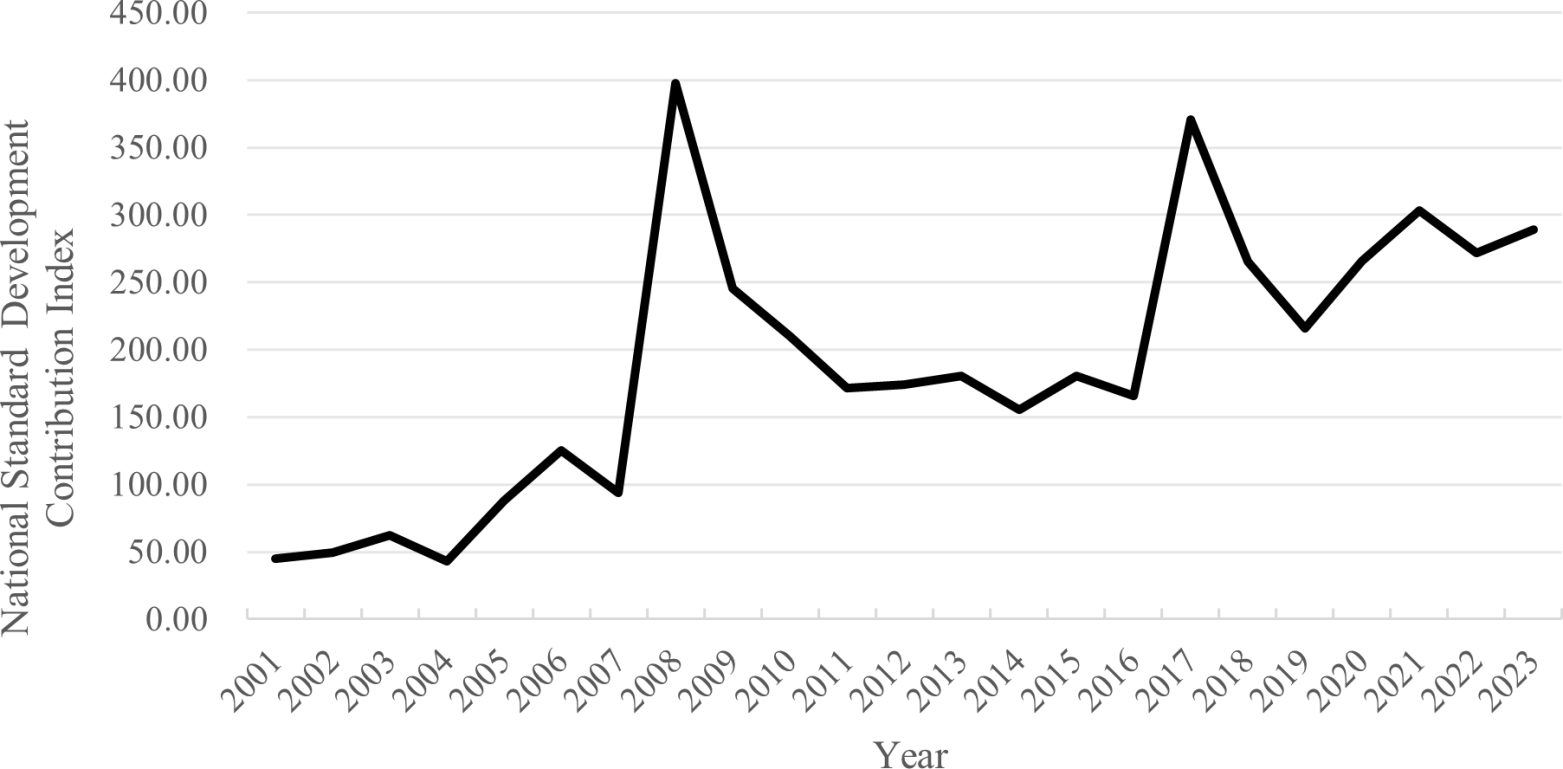
Changes in the average national standard development contribution levels in the eight comprehensive economic zones.

For the first time, this study applies machine splitting and location assignment technology to measure national standard development contribution levels across China’s eight comprehensive economic zones. The results are illustrated in Fig 1. From 2001 to 2023, the contribution levels of national standard development in these eight zones exhibited a fluctuating upward trend, increasing from 45.08 in 2001 to 289.25 in 2023, with an average annual growth rate of 23.55%. During the sample period, 2008 and 2017 were notable peak years, with the index surpassing 350.

A possible explanation for the 2008 peak is China’s increased investment in and focus on national standard development during this period, driven by domestic and international economic conditions, industrial development needs, and the government’s emphasis on standardization. In 2008, China established the goal of completing 10,000 national standards to address gaps, revise outdated standards, and combat obsolescence, resulting in a significant expansion of standard development efforts. Similarly, in 2017, as China entered a phase of high-quality development, there was an urgent need for advanced standards to drive economic progress. During this time, China emphasized the role of standards in promoting regional innovation, enhancing industrial competitiveness, and facilitating international trade, thereby prompting regions to increase investments in standard development.

During the 10th Five-Year Plan period (2001–2005), the national standard development contribution index in the eight comprehensive economic zones grew from 45.08 to 87.78, with an average annual growth rate of 23.68%. In the 11th Five-Year Plan period (2006–2010), the index increased from 125.32 to 210.14, with an average annual growth rate of 16.92%, reflecting China’s rapid economic growth and the significant expansion of national standards in industries, information technology, and infrastructure development.

During the 12th Five-Year Plan period (2011–2015), the index increased from 174.41 to 180.32, with a slower average annual growth rate of 0.85%, indicating a policy shift from quantity to quality in standard development. In the 13th Five-Year Plan period (2016–2020), the index climbed from 165.62 to 265.84, with an average annual growth rate of 15.13%, signifying China’s transition to an innovation-driven, high-quality economy in which high standards play a crucial role. During the first three years of the 14th Five-Year Plan period (2021–2023), the index declined slightly from 303.18 in 2021 to 289.25 in 2023, with an average annual decline rate of −1.15%. Notwithstanding the impact of the COVID-19 pandemic, the index remained at a relatively high level, reflecting continued investment in national standard development.

### Comparative analysis

**Fig 2:**
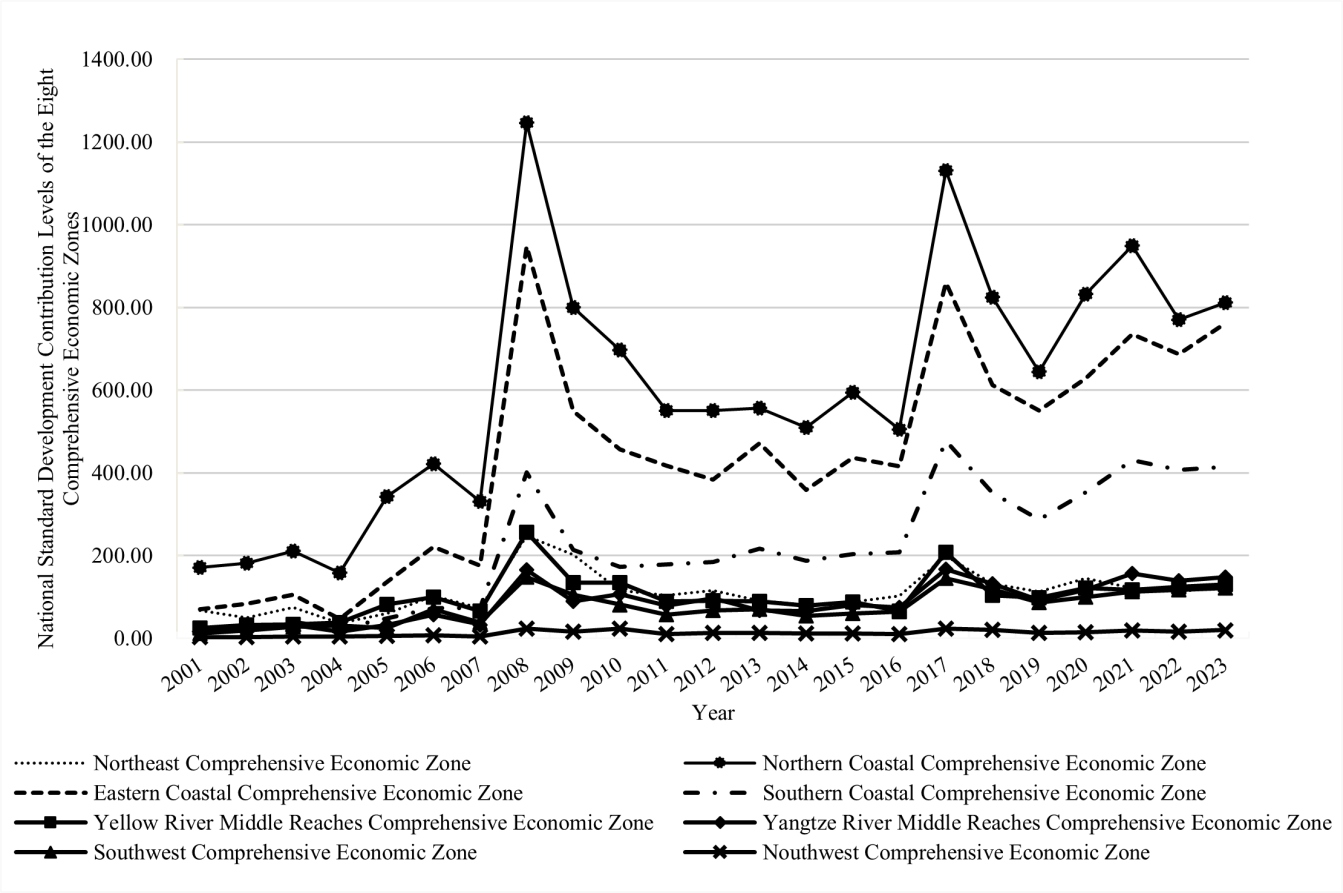
Comparison of average national standard development contribution levels across the eight comprehensive economic zones.

This section compares the national standard development contribution indices across the economic zones. As shown in Fig 2, the average contribution indices for the Northern Coastal, Eastern Coastal, and Southern Coastal zones were relatively high from 2001 to 2023, at 599.68, 439.70, and 216.73, respectively. In contrast, the Northeast, Yellow River Middle Reaches, Yangtze River Middle Reaches, Southwest, and Northwest economic zones had significantly lower average contribution indices at 111.27, 100.23, 86.64, 75.03, and 12.51, respectively. These findings indicate that coastal regions had higher national standard development contribution levels, whereas inland regions exhibited weaker performance. This disparity may be closely related to economic development levels and openness to external markets. On the one hand, the raw data suggest a positive correlation between economic development and standard development contribution levels, such that regions with higher economic activity (e.g., coastal zones) tend to make higher contributions to national standard development. In contrast, regions like the Northwest, which have lower levels of economic development, show weaker performance in standard development. On the other hand, coastal regions, having been earlier beneficiaries of China’s economic reforms and opening-up policies, have developed more mature collaborative innovation models with external entities. This has facilitated collaboration between innovation entities in these regions and external organizations, enhancing their contributions to national standard development. In contrast, inland regions have had less exposure to these external influences, resulting in lower national standard development contributions.

In terms of dynamic trends, with the exception of the relatively stable contribution levels in the Northwest economic zone, the other regions exhibit trends similar to the overall fluctuation patterns observed across the eight comprehensive economic zones. In particular, the average national standard development contribution indices for the Northeast economic zone during the 10th, 11th, 12th, 13th, and 14th Five-Year Plan periods were 57.80, 148.32, 95.59, 138.12, and 119.99, respectively, demonstrating significant fluctuations. The Northern Coastal economic zone exhibited a clear upward trend, with average contribution indices of 213.05, 699.23, 552.44, 787.60, and 843.69, respectively, over the same periods. A similar upward trend was observed in the Eastern Coastal economic zone, where indices were 88.40, 470.30, 413.87, 613.21, and 728.04, respectively. The Southern Coastal economic zone also demonstrated continuous growth, with indices of 29.88, 186.82, 194.51, 335.50, and 417.04, respectively. In contrast, the Yellow River Middle Reaches, Yangtze River Middle Reaches, Southwest, and Northwest economic zones all displayed “rise–decline–rise” patterns in their national standard development contribution indices over time.

## Section 4: Results of the Dagum Gini coefficient and its decomposition

### Overall regional disparities

**Fig 3:**
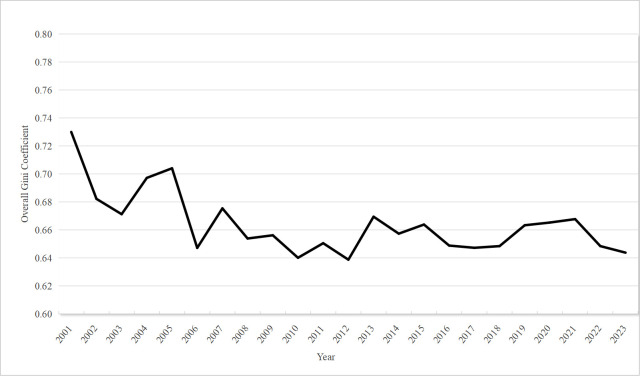
Changes in the overall gini coefficient for national standard development contribution across the eight comprehensive economic zones.

This study applies the Dagum Gini coefficient and its decomposition method to analyze regional disparities in national standard development contribution levels across the eight comprehensive economic zones, focusing on intraregional and interregional disparities. The results for overall and intraregional disparities are presented in Table 3, and Fig 3 illustrates the changes in the overall Gini coefficient for the national standard development contribution index from 2001 to 2023.

**Table 3:** Dagum Gini Coefficient and Intraregional Disparities.

Year	Overall	Northeast	Northern Coastal	Eastern Coastal	Southern Coastal	Yellow River Middle Reaches	Yangtze River Middle Reaches	Southwest	Northwest
**2001**	0.7301	0.0916	0.6457	0.4578	0.6121	0.4775	0.4709	0.5135	0.3200
**2002**	0.6823	0.1459	0.6270	0.3592	0.5738	0.4989	0.1988	0.4178	0.4000
**2003**	0.6713	0.1645	0.6249	0.3578	0.6026	0.4849	0.3564	0.3835	0.5587
**2004**	0.6973	0.2726	0.6656	0.4038	0.5769	0.4900	0.3003	0.4850	0.5224
**2005**	0.7041	0.2901	0.5772	0.2645	0.5347	0.5776	0.2178	0.4880	0.5771
**2006**	0.6472	0.3360	0.5884	0.1970	0.5998	0.3632	0.2833	0.4931	0.3505
**2007**	0.6755	0.2531	0.5788	0.2502	0.5450	0.4173	0.3350	0.5221	0.2951
**2008**	0.6539	0.2709	0.5660	0.1052	0.5535	0.3820	0.3001	0.4153	0.2977
**2009**	0.6562	0.4426	0.5686	0.0584	0.5596	0.3864	0.2325	0.3445	0.3792
**2010**	0.6401	0.4008	0.5832	0.0929	0.5472	0.2758	0.1434	0.4597	0.3990
**2011**	0.6506	0.3549	0.5602	0.0470	0.5178	0.2298	0.2355	0.4044	0.4063
**2012**	0.6388	0.3302	0.5960	0.0632	0.4969	0.3206	0.2102	0.4109	0.3880
**2013**	0.6695	0.3574	0.6082	0.0678	0.5135	0.3297	0.1443	0.3876	0.4667
**2014**	0.6574	0.3173	0.5770	0.0511	0.5082	0.3321	0.2273	0.2931	0.5150
**2015**	0.6639	0.3159	0.5697	0.0242	0.5148	0.3499	0.2191	0.4120	0.4841
**2016**	0.6489	0.3066	0.5555	0.0203	0.5025	0.3295	0.2415	0.3462	0.3156
**2017**	0.6473	0.3615	0.5738	0.0177	0.5008	0.3442	0.2576	0.3848	0.3990
**2018**	0.6485	0.2704	0.5803	0.0134	0.4939	0.3210	0.2485	0.4013	0.3592
**2019**	0.6634	0.3701	0.5655	0.0568	0.5306	0.3520	0.2369	0.4636	0.2695
**2020**	0.6653	0.3997	0.5795	0.0240	0.5290	0.3805	0.1911	0.3980	0.2787
**2021**	0.6678	0.2308	0.5823	0.0141	0.5106	0.3015	0.2329	0.4670	0.3621
**2022**	0.6485	0.2457	0.5750	0.0081	0.5229	0.2536	0.2156	0.4297	0.2116
**2023**	0.6438	0.3070	0.5629	0.0674	0.5283	0.2699	0.2020	0.3642	0.3882
**Average**	0.6640	0.2972	0.5875	0.1314	0.5380	0.3682	0.2479	0.4211	0.3889
**Average annual growth**	−0.57%	5.65%	−0.62%	−8.34%	−0.67%	−2.56%	−3.77%	−1.55%	0.88%

As shown in Fig 3, the average Gini coefficient for the period 2001–2023 was 0.6640, decreasing from 0.7301 in 2001 to 0.6438 in 2023, with an average annual growth rate of −0.57%. This finding suggests that overall regional disparities in national standard development contribution levels have been narrowing over time.

Notably, between 2004 and 2008, overall regional disparities declined significantly, which may be attributed to the lagged influence of national regional policies. The Third Plenary Session of the 16th Central Committee of the Communist Party of China, held in October 2003, emphasized regional coordinated development as part of the “Five Coordinations” initiative. This strategy aimed to gradually reverse the widening regional development gap while actively promoting the development of the Western region, revitalizing old industrial bases, supporting the rise of the Central region, encouraging the Eastern region to take the lead in development, and leveraging the advantages and enthusiasm of various regions. By improving market, cooperation, mutual assistance, and support mechanisms, the strategy led to the gradual reversal of the widening regional development gap and formation of a new pattern of mutual promotion, complementary advantages, and common development in the Eastern, Central, and Western regions.

As national regional coordinated development policies were further implemented, cooperation and exchanges in standardization efforts between regions intensified. The increasing alignment in investment and focus on national standard development contributed to greater collaboration in the drafting and implementation of standards across regions, leading to a notable reduction in overall regional disparities between 2004 and 2008.

### Intraregional disparities

Table 3 also reports intraregional disparities in national standard development contribution levels for the eight comprehensive economic zones from 2001 to 2023. The Northern Coastal and Southern Coastal zones exhibited the largest intraregional disparities, with average Gini coefficients of 0.5875 and 0.5380, respectively. In contrast, the Yangtze River Middle Reaches and Eastern Coastal zones had smaller intraregional disparities, with Gini coefficients of 0.2479 and 0.1314, respectively. These variations may be attributed to differences in technological innovation and industrial development across regions.

From an industrial value chain perspective, the Northern and Southern Coastal regions encompass industries spanning high, medium, and low levels, with an increasing trend of advanced regions (e.g., Beijing and Guangdong) transferring medium- and low-end industries to neighboring or related regions (e.g., Hebei and Hainan). Although such transfer expands the industrial scale of less developed neighboring regions, national standard development contributions are primarily driven by high-tech industries. As a result, the process of industrial transfer further increases intraregional disparities in national standard development. In contrast, the gap in industrial development quality between different areas within the Eastern Coastal and Yangtze River Middle Reaches regions is relatively small, leading to smaller intraregional disparities.

From a technological innovation perspective, standards play a critical role in promoting the industrialization of technological innovations. Regions with stronger technological innovation capacity tend to have greater demand and capability for developing national standards. The 2023 China Regional Innovation Capability Evaluation Report indicates significant intraregional disparities in technological innovation capacity within the Northern and Southern Coastal regions. In the Northern Coastal region, Beijing ranked 2^nd^, Tianjin 17^th^, and Hebei 20^th^. In the Southern Coastal region, Guangdong ranked 1^st^ and Hainan 15^th^. In the Eastern Coastal region, Jiangsu ranked 3^rd^, Zhejiang 4^th^, and Shanghai 5^th^. In the Yangtze River Middle Reaches region, Anhui ranked 7^th^, Hubei 8^th^, Hunan 9^th^, and Jiangxi 16^th^.

Regions with stronger innovation capacity dominate the development of standards, whereas those with weaker innovation capacity struggle to participate in this process, exacerbating imbalances in national standard development contributions. In contrast, the Eastern Coastal and Yangtze River Middle Reaches economic zones have smaller intraregional disparities in technological innovation capacity, leading to more balanced contributions to national standard development.

From a dynamic trend perspective, intraregional disparities in national standard development contributions have been narrowing in all regions except for the Northeast and Northwest, where disparities have widened. This finding may be attributable to increasing collaboration within industrial chains and standardization efforts in regions such as the Beijing–Tianjin–Hebei region and the Guangdong–Hong Kong–Macao Greater Bay Area, where strong knowledge spillovers have enhanced standard-setting capacities in less developed areas. In contrast, the Northeast and Northwest zones have not yet established effective mechanisms for coordinated standard development, resulting in weak interactions and limited technological spillovers. This lack of collaboration hinders synchronized standard-setting capacities, thereby increasing intraregional disparities. Additionally, some regions in the Northeast and Northwest lack motivation to engage in national standard development, and insufficient attention given to innovation further exacerbates these disparities.

### Interregional disparities

The decomposition results for interregional disparities are presented in Table 4. The largest interregional disparities were found between the Northern Coastal and Northwest zones (0.9605), the Eastern Coastal and Northwest zones (0.9384), and the Southern Coastal and Northwest zones (0.8896). The smallest disparity was observed between the Northeast and Yangtze River Middle Reaches zones, with an average value of 0.3726. Other regions showed interregional disparity values of 0.38–0.80.

**Table 4:** Decomposition results for interregional disparities.

	2001–2003		The 10th Five-Year Plan		The 11th Five-Year Plan		The 12th Five-Year Plan		The 13th Five-Year Plan		The 14th Five-Year Plan	
	Average	Average annual growth	Average	Average annual growth	Average	Average annual growth	Average	Average annual growth	Average	Average annual growth	Average	Average annual growth
**Northeast and Northern Coastal Comprehensive Economic Zones**	0.7314	0.44%	0.7084	1.58%	0.7130	1.75%	0.7443	1.50%	0.7381	1.63%	0.7676	−1.96%
**Northeast and Eastern Coastal Comprehensive Economic Zones**	0.5642	2.34%	0.4105	1.32%	0.4991	9.36%	0.6235	2.61%	0.6321	0.81%	0.7171	−0.12%
**Northeast and Southern Coastal Comprehensive Economic Zones**	0.5810	0.83%	0.5166	−3.35%	0.5463	2.12%	0.5840	1.96%	0.6140	3.05%	0.6858	−1.47%
**Northeast and Yellow River Middle Reaches Comprehensive Economic Zones**	0.3952	−2.08%	0.4972	3.01%	0.3992	0.12%	0.3630	0.67%	0.3844	3.10%	0.2905	4.84%
**Northeast and Yangtze River Middle Reaches Comprehensive Economic Zones**	0.3726	−2.84%	0.4519	−3.71%	0.4172	−2.41%	0.3369	−3.71%	0.3466	3.12%	0.2690	3.53%
**Northeast and Southwest Comprehensive Economic Zones**	0.4465	−2.97%	0.5246	−7.28%	0.4623	0.69%	0.4150	−3.04%	0.4203	2.49%	0.3859	−5.25%
**Northeast and Northwest Comprehensive Economic Zones**	0.8077	−1.12%	0.8774	−2.76%	0.8265	−6.05%	0.7775	−1.97%	0.7910	−0.39%	0.7382	−0.07%
**Northern Coastal and Eastern Coastal Comprehensive Economic Zones**	0.5741	−1.47%	0.6788	−3.67%	0.5668	−1.65%	0.5525	0.11%	0.5304	1.70%	0.5207	−1.29%
**Northern Coastal and Southern Coastal Comprehensive Economic Zones**	0.7265	−1.30%	0.8356	−2.33%	0.7538	−1.94%	0.6948	−0.06%	0.6647	0.92%	0.6553	−1.32%
**Northern Coastal and Yellow River Middle Reaches Comprehensive Economic Zones**	0.7609	−0.46%	0.7943	−2.28%	0.7224	0.55%	0.7563	1.05%	0.7675	−0.21%	0.7660	−3.15%
**Northern Coastal and Yangtze River Middle Reaches Comprehensive Economic Zones**	0.7729	−0.68%	0.8104	−0.02%	0.7853	−0.42%	0.7675	0.66%	0.7541	0.13%	0.7303	−2.24%
**Northern Coastal and Southwest Comprehensive Economic Zones**	0.8099	−0.63%	0.8404	−0.43%	0.7993	1.09%	0.8159	0.21%	0.7958	0.47%	0.7901	−3.09%
**Northern Coastal and Northwest Comprehensive Economic Zones**	0.9605	−0.10%	0.9657	−0.18%	0.9604	−0.90%	0.9576	−0.08%	0.9598	0.07%	0.9578	−0.44%
**Eastern Coastal and Southern Coastal Comprehensive Economic Zones**	0.4811	−2.24%	0.6290	−6.15%	0.5010	−4.25%	0.4139	−0.90%	0.4146	1.73%	0.4241	1.87%
**Eastern Coastal and Yellow River Middle Reaches Comprehensive Economic Zones**	0.6197	0.50%	0.5641	−5.42%	0.5264	6.52%	0.6510	0.59%	0.6839	−1.76%	0.7092	−1.27%
**Eastern Coastal and Yangtze River Middle Reaches Comprehensive Economic Zones**	0.6549	0.21%	0.5860	−1.04%	0.6640	1.13%	0.6830	0.40%	0.6822	−0.34%	0.6624	1.91%
**Eastern Coastal and Southwest Comprehensive Economic Zones**	0.6947	−0.15%	0.6429	−1.44%	0.6621	5.96%	0.7413	−0.11%	0.7152	−0.20%	0.7237	−0.67%
**Eastern Coastal and Northwest Comprehensive Economic Zones**	0.9384	0.05%	0.9149	−0.50%	0.9382	−1.08%	0.9435	−0.15%	0.9489	−0.02%	0.9515	−0.03%
**Southern Coastal and Yellow River Middle Reaches Comprehensive Economic Zones**	0.6135	0.39%	0.6172	1.36%	0.5631	−0.48%	0.5895	1.71%	0.6451	0.46%	0.6786	−2.59%
**Southern Coastal and Yangtze River Middle Reaches Comprehensive Economic Zones**	0.6070	0.31%	0.5704	−2.06%	0.6078	−2.46%	0.5995	1.20%	0.6328	1.34%	0.6357	0.20%
**Southern Coastal and Southwest Comprehensive Economic Zones**	0.6566	0.13%	0.6147	−1.67%	0.6291	2.17%	0.6684	0.59%	0.6842	1.23%	0.7066	−1.57%
**Southern Coastal and Northwest Comprehensive Economic Zones**	0.8896	0.31%	0.8395	−0.21%	0.8779	−1.71%	0.9003	0.12%	0.9172	0.37%	0.9288	−0.34%
**Yellow River Middle Reaches and Yangtze River Middle Reaches Comprehensive Economic Zones**	0.3822	−3.30%	0.5249	4.01%	0.4089	−10.80%	0.3285	6.35%	0.3340	2.41%	0.2694	−7.94%
**Yellow River Middle Reaches and Southwest Comprehensive Economic Zones**	0.4528	−2.32%	0.5690	3.43%	0.4619	−1.83%	0.3963	3.50%	0.4191	1.70%	0.3948	−9.17%
**Yellow River Middle Reaches and Northwest Comprehensive Economic Zones**	0.7859	−0.55%	0.8388	1.43%	0.8152	−5.37%	0.7613	−1.04%	0.7530	1.21%	0.7445	1.03%
**Yangtze River Middle Reaches and Southwest Comprehensive Economic Zones**	0.4033	−2.17%	0.4687	−3.17%	0.4066	−2.86%	0.3566	−0.49%	0.3921	−1.90%	0.3854	−10.00%
**Yangtze River Middle Reaches and Northwest Comprehensive Economic Zones**	0.7510	−0.41%	0.7616	−3.38%	0.7299	−5.23%	0.7348	−1.31%	0.7591	0.03%	0.7819	−1.29%
**Southwest and Northwest Comprehensive Economic Zones**	0.7305	−0.06%	0.7536	−1.15%	0.7462	−7.03%	0.6895	−1.09%	0.7306	0.09%	0.7341	−0.10%

A possible explanation for these disparities is that the Eastern, Northern, and Southern Coastal zones have been at the forefront of national standard development, benefiting from the scale effect of standardization. These regions have experienced rapid growth in standard-setting contributions while the Northwest region has lagged behind, with slower growth due to the absence of scale effects. Fig 2, which depicts evolution trends across regions, confirms significant interregional disparities in national standard development contribution levels between the Northern Coastal and Northwest economic zones, the Eastern Coastal and Northwest economic zones, and the Southern Coastal and Northwest economic zones. Conversely, both the Northeast and Yangtze River Middle Reaches economic zones have placed insufficient emphasis on national standards and have limited capacity to participate in national standard development. These regions lack relevant standardization expertise, and their technological innovation resources and capabilities remain underdeveloped. As a result, they lack the internal motivation to develop standards, leading to persistently low national standard development contributions. This explains the relatively small disparity between the two regions, with neither demonstrating substantial progress in standard-setting efforts.

The dynamic trends in interregional disparities across the eight comprehensive economic zones during the 10th to 14th Five-Year Plan periods can be categorized into four types: continuous expansion, fluctuating expansion, fluctuating contraction, and continuous contraction, as shown in Table 5.

**Table 5:** Classification of Dynamic Trends in Interregional Disparities.

Type	Number	Two zones
**Continuous expansion of interregional disparities**	6	Eastern Coastal and Northwest Comprehensive Economic Zones Southern Coastal and Northwest Comprehensive Economic Zones Southern Coastal and Southwest Comprehensive Economic Zones Eastern Coastal and Yellow River Middle Reaches Comprehensive Economic Zones Northeast and Southern Coastal Comprehensive Economic Zones Northeast and Eastern Coastal Comprehensive Economic Zones
**Fluctuating expansion of interregional disparities**	6	Yangtze River Middle Reaches and Northwest Comprehensive Economic Zones Northeast and Northern Coastal Comprehensive Economic Zones Eastern Coastal and Southwest Comprehensive Economic Zones Eastern Coastal and Yangtze River Middle Reaches Comprehensive Economic Zones Southern Coastal and Yellow River Middle Reaches Comprehensive Economic Zones Southern Coastal Yangtze River Middle Reaches Comprehensive Economic Zones
**Fluctuating contraction of interregional disparities**	12	Northern Coastal and Northwest Comprehensive Economic Zones Northern Coastal and Southwest Comprehensive Economic Zones Northeast and Northwest Comprehensive Economic Zones Northern Coastal and Yellow River Middle Reaches Comprehensive Economic Zones Southwest and Northwest Comprehensive Economic Zones Eastern Coastal and Southern Coastal Comprehensive Economic Zones Yellow River Middle Reaches and Southwest Comprehensive Economic Zones Northeast and Southwest Comprehensive Economic Zones Yangtze River Middle Reaches and Southwest Comprehensive Economic Zones Northeast and Yellow River Middle Reaches Comprehensive Economic Zones Yellow River Middle Reaches and Yangtze River Middle Reaches Comprehensive Economic Zones Northeast and Yangtze River Middle Reaches Comprehensive Economic Zones
**Continuous contraction of interregional disparities**	4	Yellow River Middle Reaches and Northwest Comprehensive Economic Zones Northern Coastal and Yangtze River Middle Reaches Comprehensive Economic Zones Northern Coastal and Southern Coastal Comprehensive Economic Zones Northern Coastal and Eastern Coastal Comprehensive Economic Zones

### Sources of regional disparities

**Fig 4:**
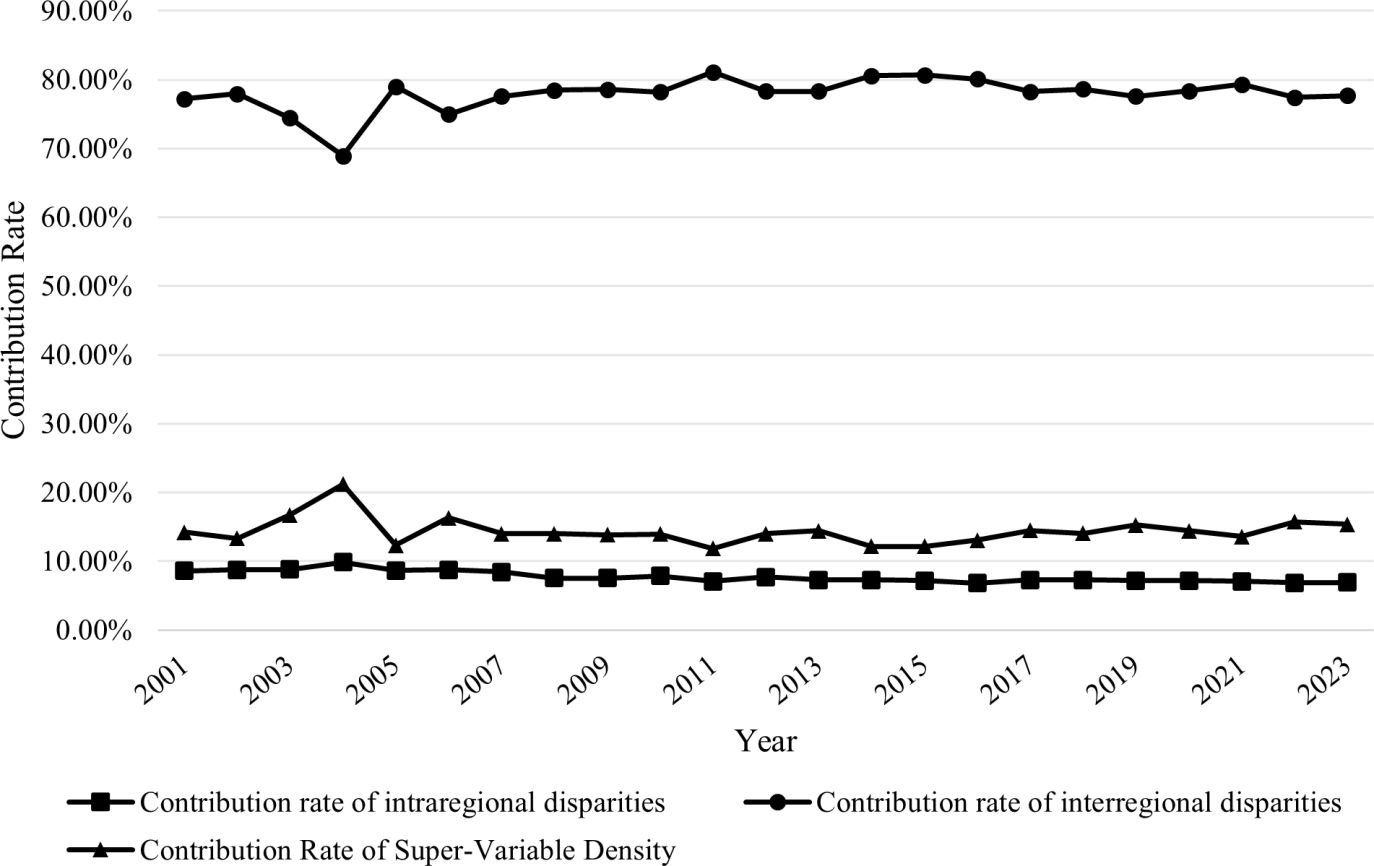
Decomposition of sources of regional disparities in national standard development contribution levels across the eight comprehensive Economic zones.

Table 6 presents the sources of regional disparities in national standard development contribution levels across the eight comprehensive economic zones, and Fig 4 illustrates the data presented in Table 6. As shown in the table and figure, the contribution rate of interregional disparities is the highest, averaging 77.89%, followed by the contribution rate of super-variable density, which averages 14.37%. The contribution rate of intraregional disparities is the lowest (7.74%), indicating that interregional disparities are the primary source of overall regional disparities in national standard development contribution levels. In terms of dynamic trends, the contribution rates of intraregional and interregional disparities and super-variable density have remained stable over time. Thus, it can be inferred that interregional disparities will continue to be the dominant source of overall regional disparities for the foreseeable future. Therefore, reducing regional disparities in national standard development contribution levels will require a sharper focus on coordinated and collaborative development across different regions.

**Table 6:** Contribution Rates of Regional Disparities in National Standard Development Contribution Levels across the Eight Comprehensive Economic Zones.

Period	Year	Intraregional		Interregional		Super-variable density	
		Source	Contribution rate	Source	Contribution rate	Source	Contribution rate
**The 10th Five-Year Plan (2001–2005)**	**2001**	0.0627	8.59%	0.5638	77.22%	0.1036	14.19%
	**2002**	0.0597	8.75%	0.5317	77.93%	0.0909	13.33%
	**2003**	0.0593	8.83%	0.4996	74.42%	0.1125	16.75%
	**2004**	0.0689	9.88%	0.4806	68.92%	0.1478	21.20%
	**2005**	0.0610	8.67%	0.5563	79.00%	0.0868	12.33%
	**Average**	0.0623	8.94%	0.5264	75.50%	0.1083	15.56%
**The 11th Five-Year Plan (2006–2010)**	**2006**	0.0566	8.75%	0.4852	74.97%	0.1054	16.29%
	**2007**	0.0571	8.46%	0.5239	77.56%	0.0944	13.98%
	**2008**	0.0494	7.55%	0.5129	78.44%	0.0916	14.01%
	**2009**	0.0496	7.55%	0.5157	78.58%	0.0910	13.87%
	**2010**	0.0503	7.86%	0.5006	78.21%	0.0892	13.93%
	**Average**	0.0526	8.03%	0.5077	77.55%	0.0943	14.42%
**The 12th Five-Year Plan (2011–2015)**	**2011**	0.0460	7.08%	0.5274	81.07%	0.0771	11.85%
	**2012**	0.0492	7.70%	0.5002	78.31%	0.0894	13.99%
	**2013**	0.0487	7.27%	0.5244	78.32%	0.0965	14.41%
	**2014**	0.0479	7.28%	0.5297	80.57%	0.0798	12.15%
	**2015**	0.0475	7.16%	0.5355	80.67%	0.0808	12.17%
	**Average**	0.0479	7.30%	0.5234	79.79%	0.0847	12.92%
**The 13th Five-Year Plan (2016–2020)**	**2016**	0.0444	6.84%	0.5198	80.10%	0.0847	13.06%
	**2017**	0.0472	7.29%	0.5065	78.25%	0.0936	14.46%
	**2018**	0.0472	7.28%	0.5099	78.64%	0.0913	14.08%
	**2019**	0.0475	7.16%	0.5145	77.56%	0.1014	15.28%
	**2020**	0.0480	7.21%	0.5215	78.38%	0.0958	14.41%
	**Average**	0.0469	7.16%	0.5144	78.59%	0.0934	14.26%
**The 14th Five-Year Plan (2020–2023)**	**2021**	0.0473	7.08%	0.5297	79.32%	0.0908	13.60%
	**2022**	0.0445	6.87%	0.5020	77.41%	0.1019	15.72%
	**2023**	0.0447	6.94%	0.5002	77.70%	0.0989	15.36%
	**Average**	0.0455	6.96%	0.5106	78.14%	0.0972	14.89%
**Overall**	**Average**	0.0515	7.74%	0.5170	77.89%	0.0955	14.37%

## Section 5: Dynamic transition characteristics of national standard development contribution levels across the eight comprehensive economic zones

### Traditional Markov chain estimation results

To further examine the dynamic transition characteristics of national standard development contribution levels across China’s eight comprehensive economic zones, this study applies traditional Markov chain estimation to construct a transition probability matrix. The results, presented in Table 7, include transition probability matrices for the entire sample period (2001–2023) and for each of the 10th, 11th, 12th, 13th, and 14th Five-Year Plan periods. The findings are as follows.

**Table 7:** Traditional Markov chain transition probability matrix for National standard development contribution levels across China’s eight comprehensive economic zones.

Period	a–1/a	Low	Lower-middle	Upper-middle	High
**2001–2023**	**Low**	0.5694	0.3472	0.0625	0.0208
	**Lower-middle**	0.1250	0.4722	0.2639	0.1389
	**Upper-middle**	0.0069	0.1042	0.3958	0.4931
	**High**	0.0000	0.0000	0.0794	0.9206
**2001–2005**	**Low**	0.6250	0.3438	0.0313	0
	**Lower-middle**	0.1563	0.5313	0.1875	0.1250
	**Upper-middle**	0.0313	0.2188	0.4063	0.3438
	**High**	0.0357	0	0.1071	0.8571
**2005–2010**	**Low**	0.7188	0.2188	0.0625	0
	**Lower-middle**	0.2500	0.4375	0.1875	0.1250
	**Upper-middle**	0	0.2813	0.4375	0.2813
	**High**	0	0	0.2857	0.7143
**2011–2015**	**Low**	0.7500	0.2500	0	0
	**Lower-middle**	0.2188	0.6250	0.1563	0
	**Upper-middle**	0	0.1563	0.6875	0.1563
	**High**	0	0	0.0357	0.9643
**2016–2020**	**Low**	0.7500	0.2500	0	0
	**Lower-middle**	0.1563	0.5938	0.2500	0
	**Upper-middle**	0	0.0625	0.6563	0.2813
	**High**	0	0	0.0357	0.9643
**2021–2023**	**Low**	0.8750	0.1250	0	0
	**Lower-middle**	0.0625	0.8125	0.1250	0
	**Upper-middle**	0	0	1	0
	**High**	0	0	0	1

First, transition characteristics vary across different levels of regional standard development contribution capabilities. For the entire sample period (2001–2023), regions with low, lower-middle, and high levels of contribution capabilities exhibit a higher probability of remaining stable than transitioning upward. In other words, regions at these levels tend to maintain their status over the long term. However, regions with upper-middle levels of contribution capabilities show a higher probability of transitioning upward, indicating that these regions, benefiting from superior innovation infrastructure and conditions, are more likely to improve their standard development capabilities over time.

Comparing different stages within the Five-Year Plan periods, regions with low, lower-middle, and upper-middle contribution capabilities have higher probabilities of maintaining stability than transitioning upward. This increased probability of stability along the main diagonal indicates that despite potential growth, these regions are less likely to make significant leaps in national standard development contribution capabilities within each five-year period. Instead, they require a longer period of accumulation to achieve major breakthroughs. Furthermore, over time, the probability of nondiagonal transitions becoming zero increases, suggesting that in recent years, regions at low, lower-middle, upper-middle, and high levels have become more stable in their national standard development contribution capabilities.

Second, a clear “club convergence” phenomenon is observed in regional standard development contribution capabilities. Over the entire period from 2001 to 2023, regions with high standard development contribution capabilities have a high probability (0.9206) of maintaining stability, with a very low probability of downward transitions. This finding indicates a club convergence phenomenon, where high-contribution regions tend to remain stable over time. Comparing different development stages within the Five-Year Plan periods, the probability of stability for high-contribution regions is 0.7143–1, further confirming the club convergence effect, which becomes more pronounced over time.

Third, the probabilities of upward and downward transitions are asymmetrically distributed. Whether examining the entire sample period or different development stages, the transition probabilities of regional national standard development capabilities are primarily concentrated in the upper right of the diagonal. This means that regions at low, lower-middle, and upper-middle levels have a significantly greater probability of transitioning upward than downward. These findings suggest that most regions tend to progress toward higher levels of national standard development contribution capabilities. This conclusion aligns with previous observations that regional contribution capabilities show an overall upward trend.

### Spatial Markov chain estimation results

Table 8 presents the spatial Markov chain transition probability matrices for the entire sample period and different development stages across the eight comprehensive economic zones. The following analysis examines spatial transition characteristics and evolutionary trends in national standard development contribution capabilities based on spatial association effects. The chi-square test results for the spatial Markov chain indicate that the Q-statistic is significant at the 1% level, confirming that spatial association factors significantly impact the dynamic transition characteristics of national standard development contribution capabilities across the eight comprehensive economic zones.

**Table 8:** Spatial Markov Chain Transition Probability Matrix for National Standard Development Contribution Levels across the Eight Comprehensive Economic Zones.

Spatial lag	a/a-1	2001–2023			
		Low	Lower-middle	Upper-middle	High
**Low**	**Low**	0.6557	0.2951	0.0492	0
	**Lower-middle**	0.2174	0.4783	0.2609	0.0435
	**Upper-middle**	0	0.0800	0.4000	0.5200
	**High**	0	0	0.1143	0.8857
**Lower-middle**	**Low**	0.5000	0.4524	0.0476	0
	**Lower-middle**	0.0980	0.5490	0.2353	0.1176
	**Upper-middle**	0	0.1351	0.4595	0.4054
	**High**	0	0	0.1429	0.8571
**Upper-middle**	**Low**	0.2778	0.3889	0.2222	0.1111
	**Lower-middle**	0.1569	0.4902	0.2157	0.1373
	**Upper-middle**	0	0.0833	0.4444	0.4722
	**High**	0	0	0.0513	0.9487
**High**	**Low**	0.6957	0.2609	0	0.0435
	**Lower-middle**	0	0.2105	0.4737	0.3158
	**Upper-middle**	0.0217	0.1087	0.3043	0.5652
	**High**	0	0	0.0526	0.9474

First, after accounting for spatial association effects, the transition probabilities of national standard development contribution capabilities across the eight comprehensive economic zones exhibit obvious spatial dependence. For the full sample period and each development stage, the transition characteristics reported in Table 8 differ substantially from those observed in the traditional Markov chain results. This indicates that the transition probabilities of national standard development contribution capabilities vary across different regions based on different levels of neighborhood environments. In other words, the transition probabilities are heavily influenced by spatial factors.

Second, there are heterogeneous effects of neighborhood environments on the improvement of regional standard development contribution capabilities. In low-level neighborhood environments, regions with low contribution capabilities tend to remain stable over time, with limited upward movement. In contrast, in high-level neighborhood environments, regions with low capabilities are more likely to progress toward higher capabilities. This suggests that high-level neighborhood environments can serve as an influencing factor in the improvement of surrounding regions’ national standard development contribution capabilities. This effect is primarily driven by competitive pressures and knowledge spillovers, which promote collaborative growth in standard development contribution capabilities in neighboring regions. Conversely, low-level neighborhood environments tend to suppress the contribution capabilities of nearby regions, limiting their growth potential.

Third, regions in upper-middle and high-level neighborhood environments are more likely to maintain their stability or achieve upward transitions. Between 2001 and 2023, regions in upper-middle and high-level neighborhood environments exhibited stability probabilities of 0.4444 and 0.3043, respectively, and upward transition probabilities of 0.4722 and 0.5652, respectively. Similarly, regions in high-level neighborhood environments demonstrated stability probabilities of 0.9487 and 0.9474.

## Section 6: Conclusions and discussion

### Research interpretations

This study examines China’s national standards from 2001 to 2023, focusing on the spatiotemporal differentiation and dynamic transition characteristics of national standard development contribution levels across China’s eight comprehensive economic zones. First, using machine splitting and location assignment technology, a national standard development contribution index was constructed for the eight economic zones from 2001 to 2023. Then, employing the Dagum Gini coefficient and its decomposition method, regional disparities in national standard development contribution levels were analyzed and their sources were identified. Finally, traditional and spatial Markov chain estimation methods were applied to determine the dynamic transition characteristics of national standard development contribution levels across the zones. The research conclusions are as follows.

### Growth of China’s national standard development contribution

From 2001 to 2023, the national standard development contribution index for China’s eight comprehensive economic zones exhibited a significant growth trend, increasing from 45.08 in 2001 to 289.25 in 2023, with an average annual growth rate of 23.55%. During the 10th, 11th, 12th, and 13th Five-Year Plans, the index showed varying degrees of positive growth, whereas in the first three years of the 14th Five-Year Plan, it exhibited negative growth. Among the regions, the Northern Coastal zone had the highest national standard development contribution index, followed by the Eastern and Southern Coastal zones; in contrast, the Northwest and Northeast zones had relatively low levels of contribution capabilities. This finding aligns with the conclusions of Zhou. [61], indicating that regions with more developed economies and greater openness, such as coastal areas, may have higher national standard development contribution levels. The regional contribution level of national standard development reflects the economic development level of the region to a certain extent.

### Narrowing regional disparities

Overall regional disparities in national standard development contribution levels across the eight zones have been reducing. The Northern and Southern Coastal zones exhibited the largest intraregional disparities. Except for the Northeast and Northwest zones, where intraregional disparities have widened, other regions have experienced a reduction. Significant interregional disparities were observed between the Northern Coastal and Northwest zones, the Eastern Coastal and Northwest zones, and the Southern Coastal and Northwest zones. In contrast, the Northeast and Yellow River Middle Reaches zones, the Yellow River Middle Reaches and Yangtze River Middle Reaches zones, and the Northeast and Yangtze River Middle Reaches zones exhibited smaller interregional disparities. The evolution of interregional disparities varies across regions, with interregional disparities being the primary driver of the overall regional gap in national standard development contribution levels. These findings address the limitations in practical application identified in previous studies [61–63]. Previous research only recognized the objective existence of regional disparities in national standard development contribution levels without quantifying the magnitude or decomposing the sources of these disparities.

### Spatial dependences of transition probability

The transition probabilities of national standard development contribution levels across the eight economic zones exhibit significant spatial dependence. Different neighborhood environments have heterogeneous effects on the improvement of regional standard development contribution capabilities. Regions in upper-middle and high-level neighborhood environments tend to maintain stability or transition upward. This finding contrasts with the conclusions of Gan [63], who determined that regions with low national standard development capabilities are constrained by developmental inertia and struggle to transition to higher levels. In contrast, this study finds that in high-level neighborhood environments, low-capability regions tend to evolve toward higher levels over time.

Thus, we can infer that high-level neighborhood environments (e.g., Beijing) can help improve the national standard development contribution capabilities of surrounding regions (e.g., Hebei and Tianjin) through competitive dynamics and knowledge spillover effects. These mechanisms foster synergistic growth in national standard development capabilities across neighboring regions. The findings of this study differ from those of previous research primarily because prior studies did not account for the impact of spatial association effects on the dynamic transition characteristics of regional national standard development contribution levels.

### Policy implications

Based on the above research conclusions, the following policy implications can be drawn.

### Promoting coordinated and collaborative resource allocation across regions

The findings of this indicate that the Eastern Coastal zone has the highest national standard development contribution index, while the Northwest and Northeast zones lag behind. Addressing this disparity requires increased investment in underdeveloped regions. On the one hand, financial and resource support should be strengthened for lagging regions by establishing special funds to support standardization projects, particularly in infrastructure construction and technology research and development. For example, dedicated standardization development funds could be established in the Northwest and Northeast zones to concentrate financial and technical resources on building national-level standardization research laboratories and technology transfer centers, thereby enhancing these regions’ standardization capabilities. On the other hand, regional standardization development plans should be formulated to clearly define each region’s focus and direction in standardization, ensuring alignment with the region’s economic and social development needs. Additionally, regional standardization databases and information-sharing platforms should be established to facilitate efficient access to standardization research outcomes and technological resources, minimizing redundant efforts and resource waste.

### Implementing coordinated regional innovation development strategies

This study shows that interregional disparities are the primary source of overall regional disparities. Therefore, guiding and allocating innovation resources is essential for fostering coordinated regional innovation development. The Eastern, Northern, and Southern Coastal zones, with their advantage of housing high-tech industries, should advance standardization in these sectors to enhance global competitiveness in technical standards. Meanwhile, the Northwest and Southwest zones, which have abundant renewable energy and eco-agriculture resources, should focus on these areas, creating distinct paths for standardization-driven innovation. Furthermore, efforts to accelerate the establishment of standardization innovation platforms should be strengthened, creating comprehensive service hubs that integrate standard development, testing, validation, and application promotion. These platforms should direct resources and efforts toward forward-looking and cutting-edge standardization research while facilitating the industrialization of standardization innovations. Establishing cross-regional standardization innovation platforms can further promote cooperation and exchanges in standardization research and application, enabling technical resource sharing and optimizing standardization efforts. This can help narrow interregional disparities and foster coordinated regional development.

### Improving the policy system for coordinated regional development

This study reveals that the transition characteristics of national standard development contribution levels across the eight economic zones exhibit significant spatial dependence. Therefore, policy frameworks must be adapted to enhance regional coordination. On the one hand, regional policy dialogue mechanisms should be established to promptly identify and resolve conflicts and inconsistencies in standardization policy implementation, ensuring policy coherence and alignment across regions. Additionally, differentiated standardization policies should be introduced to allow for flexible policy support tailored to each region’s specific conditions. The Eastern and Northern Coastal zones, as leaders in standardization, should continue to receive policy incentives to drive breakthroughs in international standardization. Meanwhile, regions with lower standardization levels, such as the Northwest and Northeast, should receive improved national policy support, including tax incentives and financial subsidies, to encourage greater engagement in standardization efforts. On the other hand, cross-regional coordination in standardization policies should be actively promoted. Successful standardization policies and best practices from leading regions should be piloted and expanded in underdeveloped regions, creating a positive cycle of standardization development. For example, the advanced standardization practices of the Eastern Coastal zone could be systematically shared with the Northwest and Northeast zones, driving improvements in standardization capacities in these regions. Additionally, the effectiveness of standardization policies should be regularly evaluated through a performance monitoring system, ensuring that standardization efforts remain on track and that timely policy adjustments can be made to enhance implementation efficiency.

### Research limitations

This study primarily examines the spatiotemporal differentiation and dynamic transition characteristics of national standard development contribution levels across China’s eight comprehensive economic zones. It relies on information on the drafting units of national standards and employs machine splitting, location assignment technology, and the Dagum Gini coefficient to quantify regional disparities in national standard development contribution levels.

However, while national standards are developed via multi-party collaboration and implemented on a nationwide scale, this study does not account for the impact of cross-regional cooperation among drafting units on regional disparities. Future research should adopt scientometric analysis to construct knowledge maps that visualize collaborative relationships among national standard drafting units. The key challenge will be efficiently constructing a large-scale knowledge map of such collaborations to extract valuable insights from these relationships.

## Supporting information

S1 Data Renamed 05c60. (XLSX)
